# Transmission stage investment of malaria parasites in response to in-host competition

**DOI:** 10.1098/rspb.2007.0873

**Published:** 2007-08-21

**Authors:** Andrew R Wargo, Jacobus C de Roode, Silvie Huijben, Damien R Drew, Andrew F Read

**Affiliations:** 1 Institutes of Evolution, Immunology and Infection Research, Ashworth Laboratories, School of Biological Sciences, University of EdinburghThe King's Buildings, West Mains Road, Edinburgh EH9 3JT, UK; 2 Western fisheries Research Center,6505 NE 65th Street, Seattle, WA 98115, USA; 3 Institute of Ecology, Ecology Building, University of GeorgiaAthens, GA 30603, USA

**Keywords:** virulence, quantitative RT-PCR, life history, transmission, gametocyte, phenotypic plasticity

## Abstract

Conspecific competition occurs in a multitude of organisms, particularly in parasites, where several clones are commonly sharing limited resources inside their host. In theory, increased or decreased transmission investment might maximize parasite fitness in the face of competition, but, to our knowledge, this has not been tested experimentally. We developed and used a clone-specific, stage-specific, quantitative PCR protocol to quantify *Plasmodium chabaudi* replication and transmission stage densities in mixed-clone infections. We co-infected mice from two strains with an avirulent and virulent parasite clone and found competitive suppression of in-host (blood-stage) parasite densities and generally corresponding reductions in transmission stage production, with the virulent clone obtaining overall competitive superiority. In response to competitive suppression, there was little evidence of any alteration in transmission stage investment, apart from a small reduction by one of the two clones in one of the two host strains. This alteration did not result in a competitive advantage, although it might have reduced the disadvantage. This study supports much of the current literature, which predicts that conspecific in-host competition will result in a competitive advantage and positive selection for virulent clones and thus the evolution of higher virulence.

## 1. Introduction

Parasite life cycles commonly involve different life stages for in-host replication and between-host transmission. Protozoan parasites, for example, often undergo clonal expansion within their host and produce morphologically distinct propagules for transmission to vectors. Likewise, parasitic worms such as cestodes typically grow and mature within their host and then release eggs into the environment. Ultimately, parasites must divide their resources between the production of transmission stages and in-host growth or replication. This represents a case of the general growth versus reproduction trade-off found in many organisms (Perrin & Sibly 1993). For pathogens, the solution to this fundamental life-history dilemma will probably impact on medically and epidemiologically relevant traits (Day 2003). For example, disease severity is frequently a consequence of densities of in-host replication stages, and infectiousness a consequence of transmission stage densities.

Malaria parasites are one of the many pathogens subject to a replication–transmission trade-off (Taylor & Read 1997). In the vertebrate host, malaria parasites replicate clonally, with a small proportion of the in-host population developing into transmission stage gametocytes. Typically, approximately 1% of parasites are gametocytes at any point in time (Taylor & Read 1997). Gametocytes cannot divide within the vertebrate host, and thus represent a loss of in-host replication, but they are the only life stage infectious to mosquitoes. Therefore, replicating parasites must ‘decide’ every cell cycle whether their progeny parasites will be capable of transmission or capable of further in-host replication; they cannot do both. Phenotypic plasticity in the rate of conversion of asexual parasites into gametocytes can be pronounced (Drakeley *et al.* 2006). For example, gametocyte investment often increases in response to environmental changes indicative of deteriorating in-host conditions, such as drug pressure and anaemia (Buckling *et al.* 1999*a*,*b*; Nacher *et al.* 2002; Paul *et al.* 2004; Reece *et al.* 2005; Ali *et al.* 2006).

There are many environmental conditions that determine how parasites should best balance the demands of replication and transmission (Gautret *et al.* 1996*b*; Buckling *et al.* 1997, 1999*a*; Eisen & DeNardo 2000; Dezfuli *et al.* 2001; Mackinnon *et al.* 2002; Osgood *et al.* 2003; de Roode *et al.* 2004, 2005*a*,*b*; Paul *et al.* 2004; Vizoso & Ebert 2005; Bell *et al.* 2006; Michaud *et al.* 2006). An important environmental factor that malaria parasites face is interactions with genetically unrelated clones in the same mammalian host (Babiker & Walliker 1997; Druilhe *et al.* 1998; Anderson *et al.* 2000; Read & Taylor 2001). There is now strong experimental and indirect epidemiological evidence of crowding effects, with individual malaria clones achieving lower densities in the presence of co-infecting clones (Daubersies *et al.* 1996; Mercereau-Puijalon 1996; Arnot 1998; Paul *et al.* 2004; de Roode *et al.* 2005*b*; Bell *et al.* 2006). The mechanism(s) responsible for competitive suppression is unclear, but could be competition for resources such as red blood cells and glucose, or apparent competition mediated by clone-transcending immune responses (Hellriegel 1992; Yap & Stevenson 1994; Gravenor *et al.* 1995; Hetzel & Anderson 1996; Buckling & Read 2001; Almogy *et al.* 2002; Råberg *et al.* 2006).

Several theorists have proposed that parasites should have phenotypically plastic life histories in response to competition (Koella & Antia 1995; Antia *et al.* 1996; West *et al.* 2001). Most obviously, clones should divert more resources into transmission to compensate for competitive suppression (Koella & Antia 1995; Antia *et al.* 1996). Such compensation is seen in response to chemotherapy and can result in treated infections transmitting as well as untreated infections (Buckling *et al.* 1997). An alternative strategy for dealing with competition might be for parasite clones to shift resources away from transmission towards replication, so as to maximize in-host numbers and competitive success. Either of these strategies might not enable a competitively suppressed clone to become the majority in the mixed infection transmission stage population, but it could significantly decrease the clone's disadvantage. Here, we test whether either of these hypotheses is correct by asking whether parasite clones alter their investment in transmission stage production (rates of gametocytogenesis) in the face of competition and, if they do, in which direction.

The existence of conditional life-history strategies involving decreased or increased transmission stage production could affect theoretical predictions about how in-host competition affects the evolution of virulence (Koella & Antia 1995; van Baalen & Sabelis 1995; Antia *et al.* 1996; West *et al.* 2001; Taylor *et al.* 2006). We return to this point in §4. For the moment, we note that life-history plasticity in response to competition has been observed in experimental studies of other pathogen systems, such as parasitic worms (Davies *et al.* 2002), sometimes suggesting increased transmission (Michaud *et al.* 2006). So far as we are aware, the issue has not been examined in microparasites, where the replication–transmission trade-off is particularly acute. In malaria, there is a broad correlation between the total proportional composition of parasite clones in the mammalian host and those in the insect vector (Taylor & Read 1998; de Roode *et al.* 2005*b*). However, the transmission investment of individual clones in mixed infections has not been studied due to technical limitations.

Determining whether individual parasite clones make facultative alterations in transmission stage investment in response to competitive suppression—and, if they do, whether it is an increase or a decrease—requires clone-specific and stage-specific quantification of parasites. Here we report the development of a quantitative reverse transcriptase PCR assay (qRT-PCR) for clone-specific quantification of early-stage gametocytes of the rodent malaria *Plasmodium chabaudi*. We used this to estimate the transmission investment of individual clones at the host population level during the acute phase of mixed infections in laboratory mice, when competitive suppression is most intense (de Roode *et al.* 2005*a*,*b*; Bell *et al.* 2006). We found evidence of resources being shifted away from transmission stage production in response to competition, but only for one of the two clones and in one of the two host strains. We found no evidence that these parasites can counter the selective effects of competition by facultative life-history plasticity.

## 2. Material and methods

### (a) Parasites and hosts

We used two genetically distinct *P. chabaudi* clones AJ and AS, originally isolated from thicket rats, *Thamnomys rutilans* (Beale *et al.* 1978). Clone AS reaches lower within-host densities, has a lower competitive ability and is less virulent than clone AJ (de Roode *et al.* 2005*b*; Bell *et al.* 2006). Hosts were six- to eight-week female C57bl/6J and CBA/ca inbred mice (herein referred to as C57 and CBA) fed on 41B maintenance diet (Harlan, UK) and drinking water supplemented with 0.05% para-amino benzoic acid to enhance parasite growth (Jacobs 1964). Mice were kept on a 12 : 12 hours light–dark cycle.

We inoculated 14 C57 and 20 CBA mice per treatment with 10^6^ AJ, 10^6^ AS or 10^6^ AS+10^6^ AJ parasites (2×10^6^ total parasites) as described elsewhere (Mackinnon & Read 1999*a*). We used equal numbers of each parasite clone for both single and mixed infections in order to compare the dynamics of each clone when it is alone versus in the presence of a competitor.

### (b) Monitoring infection dynamics

Total parasitaemia (percentage of red blood cells infected) and gametocytaemia were monitored from days 3–17 post-infection using thin blood smears of tail blood fixed in methanol and stained in 10% Giemsa. Mice were only monitored for 17 days post-infection because previous studies show that both competition and transmission decline substantially after two weeks (de Roode *et al.* 2003, 2004, 2005*b*; Bell *et al.* 2006; see §4). To determine parasite and gametocyte density, flow cytometry counts (Coulter Electronics) for red blood cell density were taken. Clone-specific parasite density of both AS and AJ in single and mixed infections was monitored using qPCR on 5 μl of whole blood taken on each day of sampling, as described elsewhere (Bell *et al.* 2006). Since qPCR relies on the quantification of parasite DNA, it cannot selectively differentiate between parasite life stages and therefore provides an estimate of the total number of parasites circulating in the blood. To accurately quantify production of transmission stages, gametocyte density was monitored for clones AS and AJ individually on each sampling day, except on days 3 and 9, using qRT-PCR as previously described (Wargo *et al.* 2006), but with the following minor modifications.

Briefly, for gametocyte qRT-PCR, 20 μl of mouse tail blood was sampled and placed in a 90 μl 1 : 2 volume mix of Ca^2+^/Mg^2+^-free PBS (Gibco) and 2×nucleic acid purification lysis solution (Applied Biosystems). To prevent possible alterations in gene expression and degradation of RNA (Fang & McCutchan 2002), all blood samples were immediately placed in lysis buffer, vortexed gently and stored at −80°C. Total RNA was then extracted on the ABI Prism 6100 machine as outlined elsewhere (Wargo *et al.* 2006). To generate cDNA, the extracted RNA underwent reverse transcription using the high-capacity cDNA archive kit (Applied Biosystems) in a 50 μl reaction containing 25 μl of RNA and 25 μl of kit reagents with the following components: 5 μl 10×RT buffer; 2 μl 25×dNTP mixture; 5 μl 10×random primers; 2.5 μl (5 U μl^−1^) multiscribe reverse transcriptase; and 10.5 μl RNase-free H_2_O. The reaction was then incubated on an MJ research DNA engine, PTC-200 at 25°C for 10 min followed by 37°C for 2 hours and 4°C hold, then stored at −80°C.

To complete gametocyte quantification, cDNA converted from RNA underwent qPCR on an ABI Prism 7000 using clone-differentiating forward primers for *P. chabaudi* clones AS (5′-AAG TTT ACC TGA GAG TAC AAA TAT AAT AGG TGT A-3′) and AJ (5′-TGA CAG TAC AAA TAT AAT AAG CGC AGT T-3′) in conjunction with a conserved reverse primer (5′-GCT GCT ATA CGT GTT ATA AAT CCT ATT ACT-3′) and TaqMan MBG probe (5′-6FAM -TGT TAT AAT TGT GTT CAC CCT ATC-3′). The qPCRs were set up for each clone at a final volume of 25 μl using 7 μl of cDNA, 900 nM forward and reverse primers, 250 nM probe and 1× concentration (supplied at 2×) TaqMan universal PCR master mix (contains AmpliTaq Gold DNA Polymerase, dNTPs with dUTP, Passive Reference, Optimized Buffer) (Applied Biosystems). All reactions were run on the ABI Prism 7000 sequence detection system under the following conditions: 50°C for 2 min; 95°C for 10 min; and then 45 cycles of 95°C for 15 s and 60°C for 1 min. All primer and probe concentrations were determined through optimizations of minimum *C*_T_ values.

The primers and probes for gametocyte qRT-PCR quantification were designed to amplify the gametocyte-specific *P. chabaudi* gene PC108476.00.0 (herein referred to as *pcs16*), the homologue of the *Plasmodium falciparum* gametocyte gene *pfs16*, known to be exclusively expressed in gametocytes. Expression of *pfs16* begins in gametocyte-committed ring stages, as one of the first events in sexual differentiation before morphological changes occur, and continues into late-stage gametocytes and gametes (Baker *et al.* 1994; Bruce *et al.* 1994; Dechering *et al.* 1997, 1999; Niederwieser *et al.* 2000; Schneider *et al.* 2004). Although little is known about *pcs16* in *P. chabaudi*, many genes, including those expressed in gametocytes, are highly conserved between *P. falciparum* and rodent malaria species such as *P. chabaudi* (Janse *et al.* 1994; Carlton *et al.* 1998, 2002; Janssen *et al.* 2001; Thompson *et al.* 2001; Waters 2002; Khan & Waters 2004; Hall *et al.* 2005).

To verify the gametocyte specificity of *pcs16*, we quantified an array of samples for both clones AS and AJ with varying levels of gametocytes using the *pcs16* assay and a separate qRT-PCR assay developed for the *P. chabaudi* gene PC302249.00.0, which is the highly conserved homologue of the *Plasmodium berghei* gene PB000198.00.0 (*P. chabaudi* primers: forward primer 5′-CAC AAT ATA GTA TAA AAG TAG GAC TTG AAA ATA ATA GTA G-3′, reverse primer 5′-GGA ATA TGG GAT ATT GTC AAA GGA TAT AC-3′, probe 5′-6FAM–TTT TCC ACT TAC AAC TCC A-3′). In *P. berghei*, the PB000198.00.0 gene has been demonstrated to have specific expression in late-stage gametocytes using GFP and promoter analyses (Khan *et al.* 2005). We found a strong correlation between the qRT-PCR counts from the *pcs16* and PC302249.00.0 assays for each clone across gametocyte densities spanning more than four orders of magnitude (AJ: *R*^2^=0.82, *F*_1,43_=131, *p*<0.001, slope=1.08±0.095, intercept=−0.23±0.28; AS: *R*^2^=0.6, *F*_1,26_=41, *p*<0.001, slope=1.14±0.18, intercept=−0.35±0.6) as well as with microscope blood smear counts across gametocyte densities spanning more than one order of magnitude (*R*^2^=0.33, *F*_1,8_=5.4, *p*=0.049, slope=1.56±0.66, intercept=−1.8±2.1). These results, combined with continued accurate quantification of both AJ and AS clones in artificial mixtures using the *pcs16* assay (correlation observed versus expected; AJ: *R*^2^=0.95, *F*_1,51_=896, *p*<0.001, slope=0.954±0.032, intercept=0.12±0.08; AS: *R*^2^=0.88, *F*_1,8_=234, *p*<0.0001, slope=0.99±0.064, intercept=−0.035±0.15), further verified gametocyte specificity and accuracy of qRT-PCR.

Standards for qRT-PCR were developed using single infections of clones AS and AJ with high levels of gametocytes and few asexual parasites. RNA was extracted and converted to cDNA as outlined above and a six-step 10-fold dilution series of cDNA was set up for each quantification run. The same clone AS and AJ standards were used throughout the experiment, creating an internal control for variation in levels of *pcs16* RNA.

To obtain asexual density estimates, we subtracted qRT-PCR RNA gametocyte counts from qPCR DNA total parasite counts. This could not be done on days 3 and 10 since gametocyte counts were not taken on these days, therefore these data points were omitted from all analyses. Less than 3% and frequently less than 0.1% of qPCR counts are due to gametocytes (see data in §3*c*), and therefore analyses assuming that qPCR data counted only asexual parasites led to identical conclusions.

### (c) Statistical analysis

All statistical analyses were carried out using general linear models in Minitab v. 14. Competitive performance was examined by comparing the performance of a clone in single infections and when a competitor was present, with explanatory variables mouse ‘strain’ (C57 or CBA), parasite ‘clone’ (AS or AJ) and infection-type ‘competition’ (clone alone or in mixed infection). For all general linear model analyses, maximal models were tested (variation in factor=clone+strain+competition+clone×strain+clone×competition+strain×competition+clone×strain×competition) and terms were dropped from the model if insignificant (*p*>0.05) until the minimal significant model was obtained. When testing for correlations, terms were added as covariates in the analysis. Where necessary, data was log (counts) or arcsine-square-root transformed (proportions) to meet assumption of normality and homogeneity of variance; likewise, gametocyte proportion data were first arcsine-square-root and then Box–Cox transformed. When examining total parasite or gametocyte production, we summed densities up to day 9, which was the latest day to which enough mice survived to compare treatment groups. To analyse the proportion of AS parasites in mixed infections, we sampled asexual density and then gametocyte density from the same mouse 1 day later, then divided the number of AS parasites by the total number of parasites present (AS+AJ). This analysis was conducted for AS only because the proportion of AJ parasites in mixed infections is simply 1−the proportion of AS parasites. Lifetime transmission potential was calculated by comparing the total number of gametocytes produced by each clone over the entire infection period of days 3–17. Dead mice were included in this analysis by filling in zeros on days after mouse death. Some mice were removed for other experiments on days 13 and 14 and could not be included in lifetime transmission analysis.

## 3. Results

### (a) Disease severity

Higher than anticipated levels of host morbidity in all infection types resulted in the euthanasia or death of several mice during the experiment. In infections composed of a single clone, 3 out of 34 (8.8%) AS-infected and 14 out of 34 (41%) AJ-infected mice died, whereas in mixed infections 11 out of 34 (32%) mice died. Thus, as in previous experiments, AJ was the more virulent clone (*Χ*_5_^2^=15.5, *p*=0.008). Approximately equal levels of host mortality were observed in CBA (18 out of 60 or 30%) and C57 mice (10 out of 42 or 24%). In most cases, mouse death occurred around peak parasitaemia, when disease symptoms were most severe. Mouse death prevented some treatment groups from being tracked for the duration of the experiment; however, all groups were followed until at least day 9 post-infection (figure 1). In spite of high host mortality, we were still able to process over 2000 qPCR and qRT-PCR samples for inclusion in the following analyses.

### (b) In-host asexual and gametocyte densities

The densities of asexual parasites and gametocytes showed similar kinetics during infections, peaking around day 8 and then trailing off (figure 1). Transmission stage production closely mirrored in-host replication for each clone, with the number of gametocytes normally two orders of magnitude lower than total parasites on any given day (figure 1).

In infections containing only a single clone, AS produced fewer asexuals and gametocytes than clone AJ (figures 1 and 2; asexuals: *F*_1,29_=18, *p*<0.001; gametocytes: *F*_1,30_=132, *p*<0.001), with the difference in asexual density between the two parasite clones being significantly greater in C57 compared with CBA mice (parasite clone×host strain interaction; asexuals: *F*_1,29_=9, *p*=0.006; gametocytes: *F*_1,29_=1.86, *p*=0.183). In general, more asexuals and gametocytes were produced in CBA compared with C57 mice (asexuals: *F*_1,29_=44, *p*<0.001; gametocytes: *F*_1,30_=78, *p*<0.001).

For both parasite clones, the presence of the other clone reduced asexual and gametocyte densities over the first 9 days of infection (figures 1 and 2). Thus, each clone experienced competitive suppression (asexuals: AS, *F*_1,32_=65, *p*<0.001; AJ, *F*_1,28_=13, *p*=0.001; gametocytes: AS, *F*_1,31_=29, *p*<0.001; AJ, *F*_1,26_=7, *p*=0.012). The level of suppression of asexuals was similar in both host strains (competition×host strain interaction: AS, *F*_1,31_=0.22, *p*=0.64; AJ, *F*_1,26_=0.76, *p*=0.39). However, competitive suppression of gametocytes was greater in CBA compared with C57 mice for clone AS (competition×host strain interaction: *F*_1,31_=6, *p*=0.019) but not for clone AJ (*F*_1,26_=3, *p*=0.088).

### (c) Transmission investment in relation to total parasite production

For both clones, there was a significant positive relationship between the total number of asexual parasites present during an infection and the total number of gametocytes (figure 3*a*,*b*; AS: *R*^2^=0.86, *F*_1,30_=16, *p*<0.001; AJ: *R*^2^=0.68, *F*_1,27_=30, *p*<0.001; all higher order interactions with the covariate were non-significant). Overall, more gametocytes were produced per asexual parasite in CBA compared with C57 mice (AS: *F*_1,30_=8, *p*=0.007; AJ: *F*_1,27_=25, *p*<0.001). We found no effect of competition on the relationship between total gametocyte and asexual parasite density for clone AJ (figure 3*b*; competition: *F*_1,25_=0.22, *p*=0.64; competition×asexual density: *F*_1,25_=2.31, *p*=0.14) or for clone AS in C57 mice (figure 3*a*). However, in CBA mice, clone AS (figure 3*a*) produced fewer gametocytes for a given asexual parasite density when in competition when compared with on its own (mouse strain×competition interaction: *F*_1,30_=7, *p*<0.012).

An alternative way of looking at transmission investment is to examine the proportion of parasites in an infection that were gametocytes as an estimate of gametocyte production rate (figure 3*c*,*d*). An average of 3% of parasites produced by clone AJ were gametocytes and 0.3% produced by clone AS (figure 3*c*,*d*), with the proportional gametocyte production being higher in CBA than in C57 mice (mouse strain main effects: AS, *F*_1,31_=26, *p*<0.001; AJ, *F*_1,26_=24, *p*<0.001). There was no effect of competition on proportional gametocyte composition of clone AJ (figure 3*d*; *F*_1,26_=0.6, *p*=0.44) or clone AS in C57 mice (figure 3*c*). In CBA mice, however, clone AS (figure 3*c*) had reduced proportional gametocyte investment in mixed compared with single infections (mouse strain×competition interaction: *F*_1,31_=7, *p*=0.011).

Thus, both covariate (figure 3*a*,*b*) and proportional analyses (figure 3*c*,*d*) revealed the same picture. Competition reduced the number of asexual parasites (figure 2) and, proportionately, the number of gametocytes of clone AJ (figures 2 and 3). This was also true for clone AS in C57 mice, but not in CBA mice, where there were disproportionately fewer gametocytes (figures 2 and 3).

To evaluate the kinetics of transmission investment, we examined the frequency of AS parasites in the asexual and gametocyte populations in mixed infections through time (number of AS parasites/(number of AS+AJ parasites)). The frequency of AS gametocytes closely tracked the frequency of AS asexuals 1 day earlier (figure 4; *R*^2^=0.48, *F*_1,58_=55, *p*<0.001). This correlation was stronger than the correlations with asexuals on the same day, or 2 or 3 days earlier, as expected from the 1-day maturation time of *P. chabaudi* gametocytes (Gautret *et al.* 1996*a*). There were no apparent changes in the relationship between the proportion of AS gametocytes and total parasites as clone AS became increasingly rare in the infections (days 7–12; figure 4).

### (d) Lifetime transmission potential

Another way to examine the fitness of an individual clone is to estimate lifetime transmission potential including dead mice in the analysis. Once a host dies, it has no transmission potential because mosquitoes do not feed on corpses, so dead mouse gametocyte densities can be set to zero. When examining the data in this way, a trend was found similar to that when analysing only the first 9 days of infection outlined previously. Over the 17 days of the experiment, clone AJ still produced more total gametocytes than AS in single-clone infections (figure 5; *F*_1,22_=82, *p*<0.001), despite high levels of mortality for mice harbouring clone AJ. Clone AS, but not clone AJ, experienced competitive suppression of lifetime gametocyte production in both mouse strains (AS: *F*_1,19_=6.8, *p*=0.018; AJ: *F*_1,24_=1.5, *p*=0.23). Total gametocyte production was also higher over the entire course of the experiment in CBA compared with C57 mice (*F*_1,22_=24, *p*<0.001).

## 4. Discussion

Life-history strategies of parasite clones during intraspecific competition have to our knowledge rarely been examined, and never in the case of medically relevant microparasites like *Plasmodium*. In this study, we measured gametocyte densities of individual clones of the rodent malaria *P. chabaudi* in mixed infections to determine whether parasites facultatively altered transmission stage production in response to competitive suppression. Despite competitive suppression of both the virulent (AJ) and less virulent clones (AS), both clones generally maintained the same level of transmission stage production throughout the infection, with the competitively superior and more virulent clone having a 10-fold higher transmission investment. Therefore, our results do not support the theoretical possibility that competitively suppressed clones allocate proportionately more resources to transmission (Koella & Antia 1995; Antia *et al.* 1996). On the contrary, in one of the mouse strains studied, the competitively inferior clone AS actually produced relatively fewer transmission stages when under competitive suppression. We note that this effect was relatively small and only found for one of the two clones in one of the two host strains. Moreover, this phenotypic plasticity did not compensate for the effects of competitive suppression: AS gametocyte densities were always reduced by the presence of AJ. Thus, we found no evidence that malaria parasites can negate the selective effects of competition by facultative life-history plasticity.

In theory, in-host competition plays a critical role in the evolution of parasite traits such as virulence, where the relatedness of clones in an infection can dramatically alter the selection on virulence. Theoretical literature largely asserts that more virulent pathogens should have an in-host competitive advantage, so that where infections are commonly composed of multiple clones, the evolution of increased virulence is expected (Levin & Pimentel 1981; Bremermann & Pickering 1983; van Baalen & Sabelis 1995; Frank 1996; Mosquera & Adler 1998; Chao *et al.* 2000; Gandon *et al.* 2001; Brown *et al.* 2002). While there is in general no logical necessity for more virulent clones to be more competitively successful (Chao *et al.* 2000; Brown *et al.* 2002; West & Buckling 2003), experimental evidence demonstrates that, at least for malaria parasites, more virulent clones are indeed more successful at in-host competition (de Roode *et al.* 2005*b*; Bell *et al.* 2006). These earlier studies of malaria looked at whether the outcome of in-host competition affected the transmission of individual clones by looking at the genetic composition of parasites in mosquitoes fed on mixed infections. There was a broad correlation between the frequency of clones in the parasite populations in the mammalian host and the insect vector (Taylor & Read 1998; de Roode *et al.* 2005*b*). To this we now add direct test of the transmission investment of individual clones in mixed infections and show that, on the whole, there is no evidence of alterations in parasite life histories in response to competition, even though these parasites are capable of phenotypic plasticity in gametocyte production in other contexts (reviewed in §1). Thus, our study supports the current literature which predicts that within-host competition will favour virulent clones and hence the spread of virulence on evolutionary time scales (Nowak & May 1994; van Baalen & Sabelis 1995; Ebert 1998; Mosquera & Adler 1998; Mackinnon & Read 1999*b*; Read & Taylor 2001; Brown *et al.* 2002; Davies *et al.* 2002; Hood 2003; Schjorring & Koella 2003; de Roode *et al.* 2005*b*; Bell *et al.* 2006).

In the present study, transmission investment responses were primarily analysed during the acute phase of infection (days 3–9) due to high levels of host death. Gametocyte production later in infections may also contribute to parasite transmission. In the case of the two *P. chabaudi* clones examined in this study, gametocyte densities have previously been observed peaking as early as day 6 post-inoculation and quickly tapering off, with substantially lower gametocyte peaks and less transmission occurring in late-stage infections (de Roode *et al.* 2003, 2004, 2005*b*). We observed similar gametocyte dynamics here for those mice tracked past day 9 (figure 1). Additionally, competition between clones was observed to be strongest during the acute phase of infection, with little competitive suppression occurring in the chronic phase (figure 1), as found previously (Bell *et al.* 2006). Therefore, we doubt that analyses beyond day 9 would have substantially altered our conclusion regarding the competitive advantage of the virulent clone, AJ. Indeed, analysis of the full 17 days of the experiment, including zero gametocyte densities for dead mice, supported the conclusion from the acute phase analyses: clone AJ produced more gametocytes than AS, despite the significantly higher level of host mortality induced by clone AJ (figure 5). Thus, the lifetime transmission potential of clone AJ was higher for clone AS, despite host deaths, confirming our conclusion that competitive interactions favour the evolution of increased virulence. Furthermore, these results demonstrate that very few gametocytes are produced in late-stage infections and potentially very little transmission is occurring in this stage.

One complexity we cannot completely rule out is the possible existence of immune-mediated apparent competition, which affects asexuals and gametocytes differently. If there is such immunity, gametocytes might be destroyed at a different rate than they are produced, thus masking underlying patterns of gametocyte investment. We consider this possibility unlikely, because we quantified gametocytes with an assay based on a gene expressed at the first step of transition from asexual to gametocyte, before there is any morphological differentiation between the two stages and so probably before any immunological differentiation (Baker *et al.* 1994; Bruce *et al.* 1994; Dechering *et al.* 1997, 1999; Niederwieser *et al.* 2000; Schneider *et al.* 2004). However, we note that even if stage-specific immune-mediated competition is masking facultative life-history shifts, these shifts are not sufficient to complicate the qualitative finding that competitive suppression of replicating parasites also results in suppressed densities of gametocytes.

Clearly, we cannot investigate all possible competition scenarios, and there may be situations where phenotypic plasticity does occur in response to the presence of other clones. For instance, in the case of superinfection, when an already infected host becomes infected again, phenotypic plasticity for transmission investment would be particularly important if the first clone to infect the host has a strong advantage (Nowak & May 1994; Hood 2003; de Roode *et al.* 2005*a*). Genotypic adjustments in commitment towards transmission may also be epidemiologically relevant when mixed infections are composed of drug-resistant and -sensitive clones. If resistant clones can increase transmission investment in competition with sensitive clones in response to environmental cues (such as drug treatment) and achieve higher fitness, this could enhance the rate of evolution of drug resistance (Buckling *et al.* 1999*b*; Hastings & Donnelly 2005). Directly testing that possibility is important, but to the extent that the results we report here generalize, we predict that there will be no such effect. Now that clone-specific quantification of the different life stages of microparasites is possible, there is considerable scope for further analyses of the epidemiological and evolutionary consequences of genetic diversity in the group-structured environments that medically important pathogens inhabit.

## Figures and Tables

**Figure 1:**
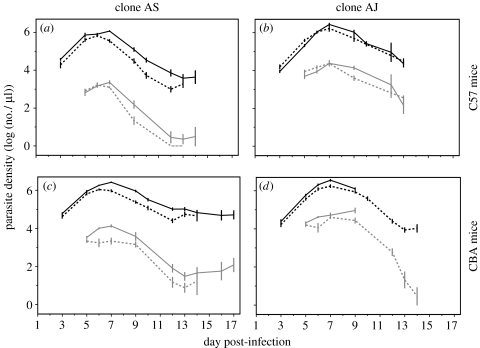
Asexual and gametocyte densities during the course of infection for parasite clones (*a*,*c*) AS and (*b*,*d*) AJ in mouse strains (*a*,*b*) C57 and (*c*,*d*) CBA. Clone performance is shown in single-clone infections (solid lines) or in the presence of the other clone (dashed lines). Gametocyte densities (grey lines) were determined by clone-specific qRT-PCR and asexual densities (black lines) by subtracting the qRT-PCR estimates from the clone-specific qPCR estimates (see text for further details). Plotted points are mean (±1 s.e.m.) of up to 19 mice; lines end when no mice were alive in respective treatment groups.

**Figure 2:**
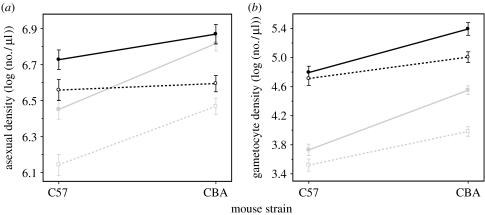
Total number of (*a*) asexual parasites and (*b*) gametocytes up to day 9 post-infection. Plotted points are least-squares means (±1 s.e.m.) for clone AJ (black lines) or AS (grey lines) in mixed (dashed lines) or single infections (solid lines), for each mouse strain. Only the 65 mice surviving to day 9 post-infection were used to determine the mean for each treatment group (CBA mice: AS alone=12, AS mixed=10, AJ alone=7, AJ mixed=10; C57 mice: AS alone=7, AS mixed=6, AJ alone=7, AJ mixed=6). In all cases, there was competitive suppression of parasites.

**Figure 3:**
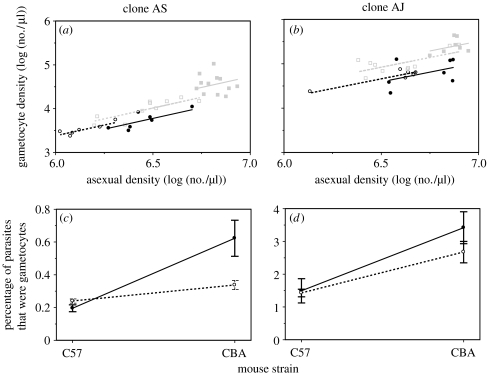
The relationship between total gametocyte and asexual parasite production. (*a*,*b*) Total gametocyte production was compared with total parasite production for each mouse alive on day 9 post-infection. Minimal significant model regression fit lines (slopes were not significantly different; see §2 for maximum model) are given for clones (*a*) AS and (*b*) AJ in single (solid lines) and mixed infections (dashed lines) in C57 (black circles) and CBA mice (grey squares). (*c*,*d*) We also compared the percentage of parasites that were gametocytes. Plotted points are mean percentage (±1 s.e.) for clones (*c*) AS and (*d*) AJ in single (solid line) and mixed infections (dashed line). Overall, we found that significantly more gametocytes were produced for a given parasite density in CBA compared with C57 mice. Additionally, clone AS had higher proportional gametocyte production in single versus mixed infections of CBA mice.

**Figure 4:**
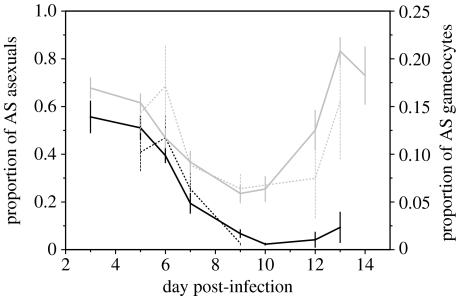
Proportion of parasites in mixed infections that were clone AS. The proportion of AS asexuals (left *y*-axis, solid lines) and gametocytes (right *y*-axis, dashed lines) were tightly correlated through time (*R*^*2*^=0.48, *F*_1,58_=55, *p*<0.001) in both C57 (black lines) and CBA mice (grey lines). Standard error bars (±1 s.e.) are given, with values based on days in which both clones were detected via qPCR or qRT-PCR, and two or more mice were alive in respective treatment groups.

**Figure 5:**
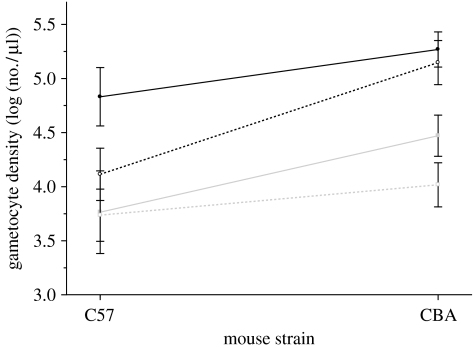
Lifetime transmission potential. Plotted points are least-squares means of total gametocytes produced up to day 17 post-inoculation (±1 s.e.m.) for clone AJ (black lines) or AS (grey lines) in mixed (dashed lines) or single infections (solid lines), for each mouse strain. After host death, gametocyte densities were taken as zero. Some mice were removed for a different experiment on days 13 and 14 and were excluded from the analysis. Sample sizes were as follows. CBA mice: AS alone=8, AS mixed=7, AJ alone=11, AJ mixed=7; C57 mice: AS alone=2, AS mixed=5, AJ alone=4, AJ mixed=5. In all cases, there was competitive suppression of gametocytes, and clone AJ produced more gametocytes over the course of infection than clone AS, despite inducing higher mortality.
